# Quercetin-Loaded Zein/Carboxymethyl Chitosan Nanoparticles: Preparation, Characterization and Evaluation for Enhanced Stability and Antioxidant Activity

**DOI:** 10.3390/molecules31020288

**Published:** 2026-01-13

**Authors:** Haiqi Yu, Wanjun Chen, Yuhong Su, Mengdie Mo, Fei Yu, Xiaodong Chen

**Affiliations:** 1 Guangxi Key Laboratory of Special Biomedicine, School of Medicine, Guangxi University, Nanning 530004, China; 2 Suzhou Key Laboratory of Green Chemical Engineering, School of Chemical and Environmental Engineering, College of Chemistry, Chemical Engineering and Materials Science, Soochow University, Suzhou 215123, China

**Keywords:** zein, carboxymethyl chitosan, quercetin, stability, antioxidant activity

## Abstract

As a natural flavonoid compound, quercetin possesses excellent antioxidant, anti-inflammatory and anti-atherosclerotic activities. However, the poor water solubility and sensitivity to the environment severely limit the application of quercetin. Initially, quercetin-loaded zein/carboxymethyl chitosan nanoparticles (ZCQ NPs) were prepared using an anti-solvent precipitation method. The fabricated ZCQ NPs exhibited a small particle size and polydispersity index (PDI). The ZCQ NPs had a negative zeta potential with an absolute value of 41.50 ± 1.76 mV. ZCQ NPs could remain highly stable against light, heat and ion strength. In addition, ZCQ NPs maintained good monodispersity and displayed minimal changes in particle size under long-term storage conditions. Additionally, a superior antioxidant capacity of ZCQ NPs was also observed in the free radical and reactive oxygen species (ROS) scavenging study compared to that of free quercetin. All these results of this study suggest that ZCQ NPs could serve as an effective drug delivery system for encapsulating and delivering quercetin.

## 1. Introduction

Quercetin (Quer), a natural flavonoid compound, is isolated from commonly consumed fruits and vegetables [1]. As a natural plant polyphenol, Quer possesses various bioactive activities, including anti-inflammatory, antimicrobial and anti-atherosclerotic properties [2]. Additionally, it was reported by earlier studies that Quer is an excellent antioxidant [3]. However, the widespread application of Quer in pharmaceutical and food industries is still limited, which is mainly attributed to its low water solubility and instability to environmental stresses, such as UV light and heat [4]. In recent years, nanoparticles have been employed to encapsulate Quer to overcome its aforementioned drawbacks. Nowadays, nanomedicine delivery systems based on plant proteins have been widely studied, owing to their high encapsulation capacity and unique structural properties [5,6]. Among these, zein-based nanoparticles have gained widespread attention.

Zein, a natural protein extracted from the endosperm of corn, is the major storage protein of corn [7]. Due to its low cost, excellent biocompatibility and biodegradability, zein is widely utilized in the food industry [8,9]. More than half of the hydrophobic amino acids are distributed on the surface of zein, which makes it insoluble in water but soluble in high-concentration ethanol aqueous solution (60–90%) [10]. In addition, the unique amino acid distribution also endows zein with amphiphilicity. This enables zein to self-assemble into nanoparticles via the anti-solvent precipitation method for Quer encapsulation [11]. Unfortunately, the application of zein nanoparticles (ZNPs) is hindered due to their tendency to aggregate in solutions [12]. Moreover, ZNPs often present poor stability when exposed to high temperatures and ionic solutions [13]. Notably, it would be a feasible solution to add a polymer as a stabilizer. Recently, numerous synthetic polymers have been reported for stabilizing ZNPs. Nevertheless, these synthetic polymers produced underlying toxicity in the preparation process and entailed an economic burden. Fortunately, CMCS could tackle these problems effectively.

Carboxymethyl chitosan (CMCS), as a classic polysaccharide, is a derivative of natural chitosan [14]. CMCS contains groups that interact with water, including amines and carboxyl groups, and also contains glucose backbones that do not interact with water. This amphiphilic property allows it to attach to zein surfaces when conditions are acidic [15]. Moreover, the excellent water solubility of CMCS could significantly improve the hydrophilicity of ZNPs. Additionally, the high safety and low toxicity that CMCS shows indicate that it provides an ideal means for stabilizing ZNPs [16].

In this study, ZCQ NPs were prepared via an anti-solvent precipitation method. The ZCQ NPs were characterized in terms of average diameter, PDI, zeta potential, encapsulation efficiency (EE) and load capacity (LC). Moreover, the morphology of the nanoparticles was observed using TEM. FT-IR was applied to study the driving forces of ZCQ NPs. In addition, X-ray diffraction (XRD) was conducted to explore the crystalline state of Quer after ZCQ NPs formation. Furthermore, the light stability, heat stability, ion strength stability and storage stability of the ZCQ NPs were investigated. An in vitro drug release test under simulated gastrointestinal conditions was carried out to measure the release behavior of ZCQ NPs and the sustained-release profile was verified. Specifically, the influence of nanoparticles on the drug’s endocytosis efficiency was explored through the implementation of cellular uptake assays. Additionally, an intracellular-ROS scavenging experiment was carried out to investigate the intracellular antioxidant capacity of ZCQ NPs.

## 2. Results and Discussion

### 2.1. Assembly Mechanism of ZCQ NPs

**Figure 1:**
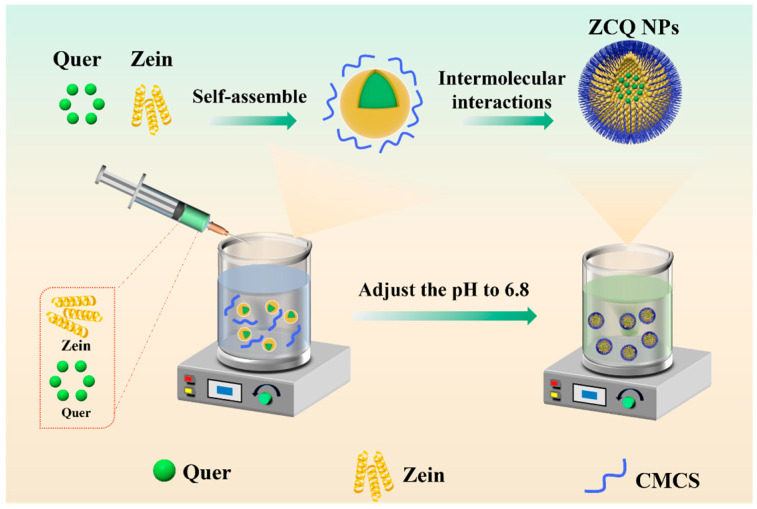
Schematic illustration of the formation of ZCQ NPs.

In this work, ZCQ NPs were synthesized via an anti-solvent precipitation method (Figure 1). Briefly, zein and Quer were first co-dissolved in an ethanol aqueous solution (80%, *v*/*v*), which was subsequently dropped into an aqueous phase. After that, the solubility of zein in the mixed solvent significantly decreased, resulting in the occurrence of supersaturation. The zein would be induced to nucleate when the supersaturation of the solution surpassed a threshold. Meanwhile, the zein would self-assemble into nanoparticles (ZNPs), while the co-dissolved Quer was encapsulated into ZNPs via hydrophobic interactions. Lastly, the pH value of the system was adjusted to 6.8 with acetic acid to induce the self-aggregation of CMCS. And the glucose–carbon skeleton of CMCS would interact with the hydrophobic amino acids of zein under the intermolecular interactions (e.g., hydrophobic interaction). Finally, ZCQ NPs were successfully synthesized.

### 2.2. Preparation and Characterization of ZC NPs

**Figure 2:**
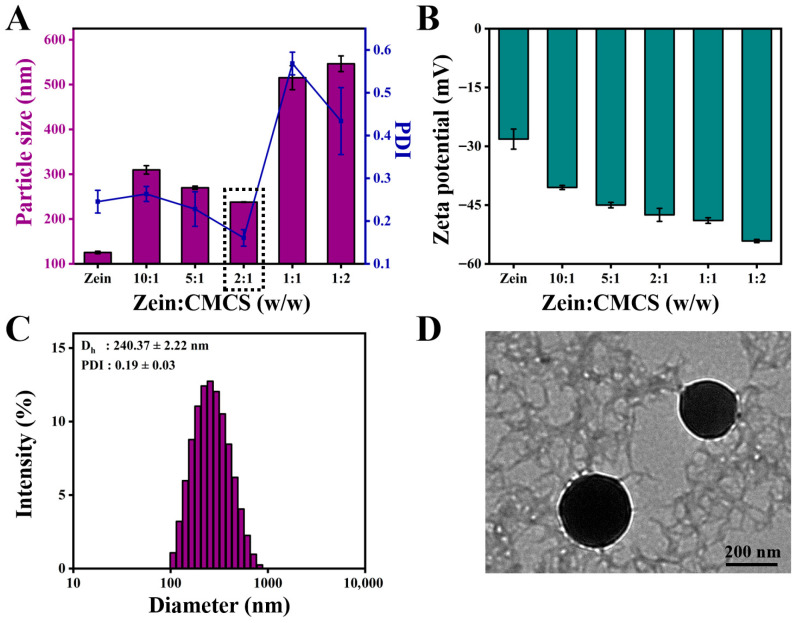
Effect of zein to CMCS mass ratios on (**A**) particle size, PDI and (**B**) zeta potential of ZC NPs. (**C**) Particle size distribution of ZCQ NPs. (**D**) TEM image of ZCQ NPs.

Subsequently, the mean diameter, PDI, and zeta potential were chosen as the elements to assess the effect of the addition of CMCS on the formation of ZC NPs. As shown in Figure 2A, the mean diameter of ZC NPs was obviously larger than that of zein, which may be closely related to the surface adsorption of CMCS on zein. When the mass ratio of zein to CMCS increased from 10:1 to 2:1, the particle size of ZC NPs decreased from 309.70 ± 9.44 nm to 237.77 ± 0.55 nm. The decrease in particle size might be due to the enhanced interaction between zein and CMCS [17]. However, the mean size of ZC NPs increased gradually as the amount of CMCS continued to increase, which was likely due to excessive CMCS adsorbed on the surface of zein. It was noteworthy that the ZC NPs exhibited the minimum particle size and PDI when the mass ratio was 2:1. As shown in Figure 2B, the zeta potential of ZC NPs exhibited negative values, which were significantly lower than those of ZNPs (without CMCS). This phenomenon further confirmed the successful adsorption of CMCS onto the surface of zein [18]. Meanwhile, when the mass ratio of zein to CMCS reached 2:1, the zeta potential was −47.50 ± 1.66 mV, which indicated that the prepared ZC NPs were a relatively stable system [19]. Based on the above results, a mass ratio of 2:1 was selected as the optimum one for Quer encapsulation.

### 2.3. Characterization of ZCQ NPs

**Figure 3:**
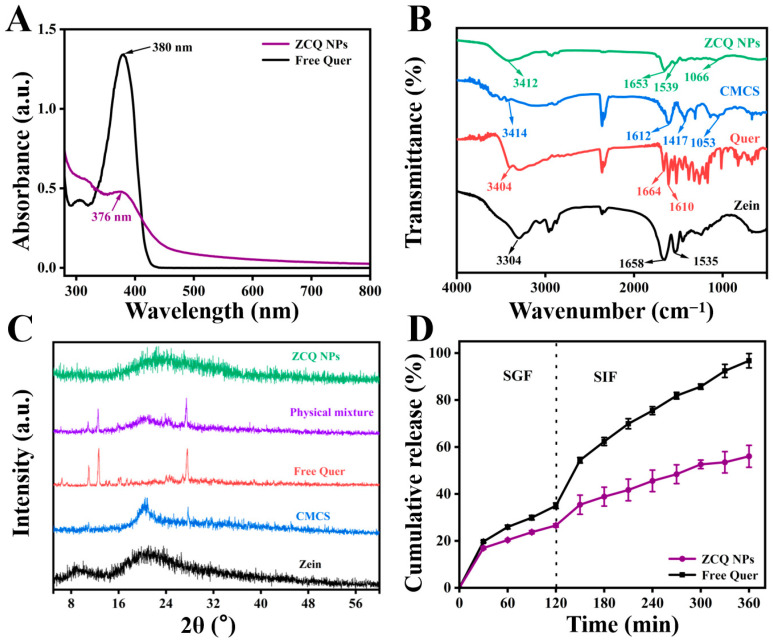
(**A**) UV-vis absorption spectra of free Quer and ZCQ NPs. (**B**) FT-IR spectra of zein, Quer, CMCS and ZCQ NPs. (**C**) XRD patterns of zein, CMCS, Quer, physical mixture and ZCQ NPs. (**D**) In vitro release profiles of ZCQ NPs and free Quer.

The mean particle size and PDI of ZCQ NPs are shown in Figure 2C. Obviously, ZCQ NPs possessed a relatively small particle size (240.37 ± 2.22 nm) and PDI (0.19 ± 0.03). The results of dynamic light scattering (DLS) showed that the incorporation of Quer had no significant effect on the particle size of the nanoparticles. The zeta potential of ZCQ NPs was −41.50 ± 1.76 mV. The larger absolute value of the zeta potential indicated that ZCQ NPs had better anti-aggregation ability. As shown in Figure 2D, the ZCQ NPs exhibited a regular spherical shape with a diameter of around 240 nm, which was consistent with the results of the DLS measurement. In addition, the EE and LC of ZCQ NPs were 83.41 ± 2.11% and 4.26 ± 2.52%, respectively. The high EE value indicated that most of Quer was encapsulated effectively. The UV spectrum of ZCQ NPs (Figure 3A) showed an absorption peak similar to that of free Quer at 376 nm, which indicated the successful encapsulation of Quer [20].

### 2.4. FT-IR Analysis

FT-IR was used to explore the interactions between zein, Quer and CMCS. The FT-IR spectra of zein, Quer, CMCS and ZCQ NPs are shown in Figure 3B. When it comes to the spectrum of zein, the absorption peak at 3304 cm^−1^ was attributed to the stretching vibration of O-H, while the peaks at 1658 cm^−1^ and 1535 cm^−1^ belonged to the amide I band and amide II band, respectively [21]. Obviously, the amide I band of zein shifted to 1653 cm^−1^, while the amide II band of zein shifted to 1539 cm^−1^ after the formation of ZCQ NPs. Such phenomena were possibly due to the formation of hydrogen bonds between the carboxyl groups in CMCS and the carbonyl group in the amide bond of zein [22]. Meanwhile, the existence of hydrogen bonding could also be proved by the noticeable shift (from 3304 cm^−1^ to 3412 cm^−1^) in the O-H vibration peak of ZCQ NPs [23]. In addition, the characteristic absorption peak of the -COC- group in CMCS was observed at 1053 cm^−1^, and a similar absorption was found at 1066 cm^−1^ in ZCQ NPs, indicating the successful adsorption of CMCS. Furthermore, most of the characteristic peaks of Quer disappeared after the formation of ZCQ NPs, confirming the successful encapsulation of Quer [24]. In addition, since both zein and Quer are hydrophobic materials, hydrophobic interactions could potentially play a role as one of the driving forces behind nanoparticle formation [25].

### 2.5. XRD Patterns

It is widely recognized that the crystalline state is closely associated with the stability and solubility of hydrophobic drugs. Therefore, XRD tests were carried out to examine how the formation of ZCQ NPs influences the crystalline characteristics of Quer. The XRD patterns of zein, Quer, CMCS, the physical mixture and ZCQ NPs are presented in Figure 3C. Zein exhibited two relatively broad and flat peaks at 9.31° and 19.35°, indicating the amorphous nature of the protein. The XRD pattern of Quer presented prominent characteristic peaks at 6.47°, 10.94°, 13.95°, 14.50°, 18.14°, 24.99° and 27.63°, manifesting the highly crystalline nature of Quer [26]. Similar crystalline peaks were also observed in the physical mixture, indicating that the crystallinity of Quer could not be changed by physical mixing alone. However, the crystal peaks of Quer disappeared in the pattern of ZCQ NPs, which indicated that Quer changed from a highly crystalline state to an amorphous state [27].

### 2.6. Release Profiles

The release profiles of free Quer and ZCQ NPs under simulated gastrointestinal conditions are shown in Figure 3D. During 2 h of incubation in SGF, 34.97 ± 1.18% of free Quer was released. A total release of over 95% of free Quer was detected after the entire simulated-digestion period. In contrast, ZCQ NPs exhibited a relatively slow release rate during the entire digestion period. Only 56.03 ± 4.68% of Quer was released from ZCQ NPs at the end of the simulated digestion, which was ~30% less than that of free Quer. It was evident that the encapsulation of Quer within ZCQ NPs leads to a controlled-release effect in the release rate of Quer. The release behavior of ZCQ NPs occurred in a slow and sustained manner, providing better protection for the encapsulated Quer. It has been reported that the sustained-release behavior contributed to improving drug bioavailability because it prevented digestive enzyme degradation [28]. In addition, the controlled-release phenomenon of ZCQ NPs might be caused by the intermolecular interaction that exists between the Quer and ZC NPs, resulting in a compact structure of ZCQ NPs [29]. Additionally, ZC NPs could act as a protective layer, which could prevent the internal Quer from directly interacting with the digestive fluid [30]. Lastly, hydrophilic CMCS could delay the degradation rate of zein by enzymes in the digestive fluid [31]. All the above results demonstrate that ZC NPs could act as a qualified delivery system for hydrophobic active drugs.

### 2.7. Stability Investigation

#### 2.7.1. Ionic Strength Stability

**Figure 4:**
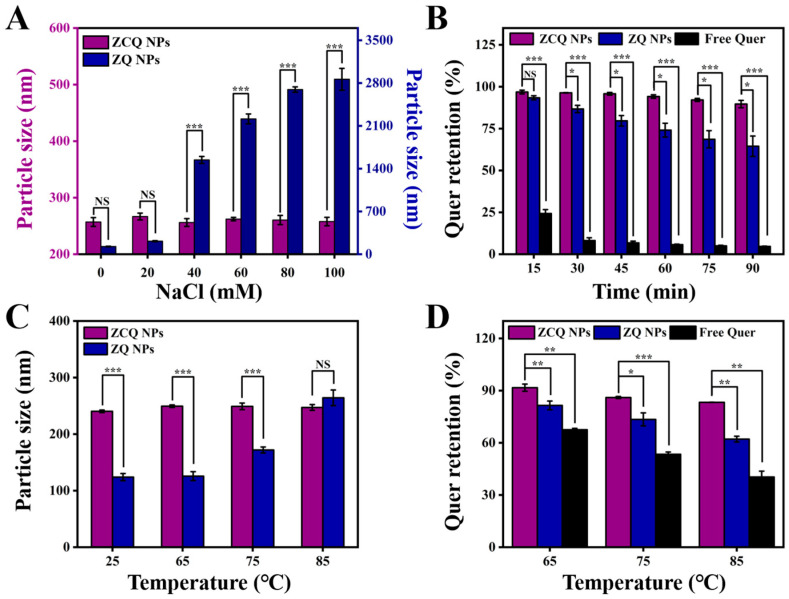
(**A**) Particle size of ZCQ NPs and ZQ NPs after incubation with different concentrations of NaCl. (**B**) Quer retention of ZCQ NPs, ZQ NPs and free Quer during 90 min of UV irradiation. (**C**) Particle size of ZCQ NPs and ZQ NPs after incubation at 25, 65, 75 and 85 °C for 30 min. (**D**) Quer retention after incubation at 65, 75 and 85 °C for 30 min. (NS: *p* > 0.05, * *p* < 0.05, ** *p* < 0.01, *** *p* < 0.001).

ZCQ NPs would experience various ionic strengths during production, transportation and oral processes [32]. Therefore, it was essential to investigate the impact of ionic strength on the stability of ZCQ NPs. The impact of ionic strength on the average diameter of ZCQ NPs and ZQ NPs is presented in Figure 4A. Evidently, ZQ NPs displayed a marked growth in particle size when the NaCl concentration increased from 0 mM to 100 mM. This effect might be connected with the poor stability of zein, as it tends to aggregate when exposed to high ionic strength. Conversely, the particle size of ZCQ exhibited no major change. Small changes in particle size meant that the ZCQ NPs were fairly stable at the tested concentration. The enhanced salt resistance of ZCQ NPs could result from the high zeta potential of ZCQ NPs, which endows the nanoparticles with strong electrostatic repulsion to resist the effects imposed by the external salt solution [33].

#### 2.7.2. Photostability of Quer

The application of Quer is severely restricted by its sensitivity to UV light. Therefore, it is necessary to study the photostability of ZCQ NPs under UV light. As depicted in Figure 4B, free Quer exhibited low stability under UV exposure; the retention rate of Quer dropped to less than 10% after 1.5 h. In contrast, 64.46% of the Quer remained in the ZQ NPs after 1.5 h of UV irradiation, indicating that encapsulation in zein could provide protection for Quer. It is essential to point out that the retention rate of Quer in ZCQ NPs was 89.63 ± 2.22%, which was 1.39-fold and 19.32-fold higher than that of ZQ NPs and free Quer, respectively. These findings are consistent with the work of Khan, Muhammad Aslam et al. [34]. Such results revealed that ZCQ NPs exhibited greater photochemical stability, which might be linked to the following factors: on the one hand, ZC NPs supplied a protective shell for the encapsulated Quer [35]; on the other hand, the aromatic groups and double bonds in zein and CMCS could absorb a significant portion of UV light, which could reduce the degradation of Quer [36].

#### 2.7.3. Thermal Stability

Nanosystems might be exposed to high temperatures during the production and transportation processes. Therefore, the stability of free Quer, ZQ NPs and ZCQ NPs at different temperatures was investigated. As shown in Figure 4C, the particle size of ZQ NPs exhibited a significant increase (from 124.03 nm to 264.27 nm) with the elevation of the water bath temperature. In contrast, the particle size of ZCQ NPs did not present obvious fluctuation under the test temperatures, which suggested that ZCQ NPs possessed increased anti-aggregation properties under thermal treatment. Meanwhile, the retention rates of ZCQ NPs, ZQ NPs and free Quer after heating were also measured. As can be seen in Figure 4D, the retention rates of free Quer were 67.53%, 53.43% and 40.36% after 30 min of heating at 65, 75 and 85 °C, respectively. However, the retention rates of ZCQ NPs were 91.69%, 86.05% and 83.26%, respectively, which are obviously higher than those of ZQ NPs (the retention rates of ZQ NPs were 83.51%, 72.46% and 62.13% after being inducted at 65, 75 and 85 °C, respectively). This outcome agreed well with the work of Sun et al., which demonstrated that the synthesized nanoparticles could exert superior protective effects on Quer against thermal stress [37]. A possible reason for the increased stability was that Quer had been encapsulated within the hydrophobic cavities of ZCQ NPs, which reduced the impact of external temperatures on Quer [38].

#### 2.7.4. Storage Stability and Redispersion of ZCQ NPs

**Figure 5:**
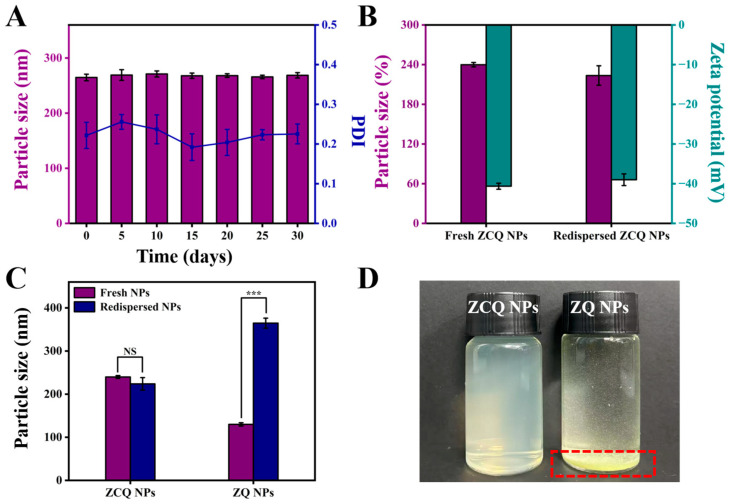
(**A**) Particle size of ZCQ NPs after 0, 5, 10, 15, 20, 25 and 30 days of storage. (**B**) Particle size and zeta potential of fresh and redispersed ZCQ NPs. (**C**) Particle size of fresh and redispersed ZCQ NPs and ZQ NPs. (**D**) The visual appearance of redispersed ZCQ NPs and ZQ NPs. (NS: *p* > 0.05, *** *p* < 0.001).

The stability during the storage period is crucial to nanosystems. The ZCQ NPs were stored at 4 °C for 30 days to assess the storage stability. The mean particle size measurements at different time points are shown in Figure 5A. During the storage period, ZCQ NPs exhibited slight changes in particle size with the PDI below 0.25. The zeta potential of ZCQ NPs at 30 days of storage was −35.67 ± 2.17 mV, implying that the ZCQ NPs were relatively stable. In summary, the ZCQ NPs could maintain good stability within 30 days of storage.

The redispersibility of nanosystems in water is of paramount importance for their applications in both the food and pharmaceutical industries [39]. In this study, the freeze-dried powders of ZCQ NPs and ZQ NPs were redispersed in deionized water, and the particle diameter and zeta potential were measured. As shown in Figure 5B, the redispersed ZCQ NPs exhibited no significant change in particle size compared to freshly prepared ZCQ NPs (from 239.90 nm to 223.83 nm). In addition, the zeta potential also maintained a high absolute value (from 40.63 mV to 39.00 mV). DLS measurements indicated that the redispersed ZCQ NPs retained high stability. However, as depicted in Figure 5C, the particle size of redispersed ZQ NPs increased clearly compared to the freshly prepared ones (from 129.97 nm to 364.60 nm). Furthermore, freeze-dried ZQ NPs could not be uniformly dispersed in the aqueous phase (Figure 5D), whereas the redispersed ZCQ NPs remained a clear and homogeneous solution. This phenomenon could be attributed to the strong hydrophobicity of zein, while the adsorption of CMCS on the surface of zein significantly enhanced the water redispersibility of zein [40]. All results demonstrated that freeze-drying did not compromise the water redispersibility of ZCQ NPs, providing a new strategy in the storage and transportation of nano-formulations.

### 2.8. Cellular Uptake Study

**Figure 6:**
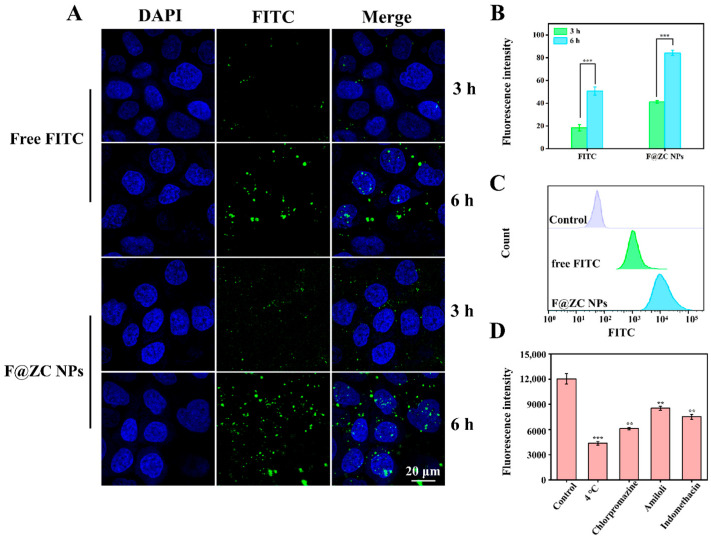
(**A**) CLSM images of Caco-2 cells incubated with free FITC or F@ZC NPs. (**B**) Quantification of fluorescence of FITC obtained in CLSM images. (**C**) Flow cytometry analysis of Caco-2 cells’ cellular internalization of free FITC and F@ZC NPs at 6 h. (**D**) Effect of different inhibitors on cellular uptake of F@ZC NPs. (** *p* < 0.01, *** *p* < 0.001).

CLSM was used to observe the cellular uptake of Caco-2 cells treated with free FITC and F@ZC NPs. As presented in Figure 6A, the Caco-2 cells showed time-dependent uptake behavior under the treatment with free FITC and F@ZC NPs. In addition, the free FITC group exhibited weak green fluorescence, indicating that only a small amount of FITC was internalized by Caco-2 cells. It is worth mentioning that after 6 h of incubation, Caco-2 cells treated with F@ZC NPs exhibited increased fluorescence intensity compared to free FITC. In addition, the quantitative analysis of the green-fluorescence intensity revealed a significant 1.66-fold increase in the F@ZC NPs group compared to in the free FITC group following 6 h of treatment (Figure 6B). The results of the flow cytometry in Figure 6C also show that the fluorescence intensity of F@ZC NPs was significantly higher than that of free FITC, which was consistent with the CLSM detection results. All the above results proved that F@ZC NPs had a higher cellular-uptake efficiency than did free FITC. The enhancement of cellular-uptake capability may be attributed to the improved dispersibility of drugs encapsulated in ZC NPs. This could allow more hydrophobic drugs to reach the cell membrane surface and be effectively taken up by the cells [41]. Furthermore, it was demonstrated by previous reports that nanoparticles with smaller particle sizes (less than 300 nm) were prone to being endocytosed by intestinal cells, which was consistent with the obtained result in this study [42].

### 2.9. Endocytosis Mechanisms of Nanoparticles

Chlorpromazine (inhibitor of clathrin-mediated endocytosis), amiloride (inhibitor of macropinocytosis), and indomethacin (inhibitor of caveolae-dependent endocytosis) were applied to investigate the cellular uptake mechanism of F@ZC NPs by Caco-2 cells [43,44]. As presented in Figure 6D, the 4 °C treatment group exhibited the lowest relative fluorescence intensity, suggesting that the uptake process of nanoparticles was energy-dependent. Notably, the chlorpromazine group displayed a markedly lower fluorescence intensity compared with those groups treated with the other two inhibitors, which demonstrated that clathrin-mediated endocytosis served as the predominant route for F@ZC NPs internalization. In addition, reduced fluorescence intensity was also observed in both amiloride and indomethacin treatment groups, which supported the participation of macropinocytosis and caveolae-dependent endocytosis.

### 2.10. Antioxidant Activity

**Figure 7:**
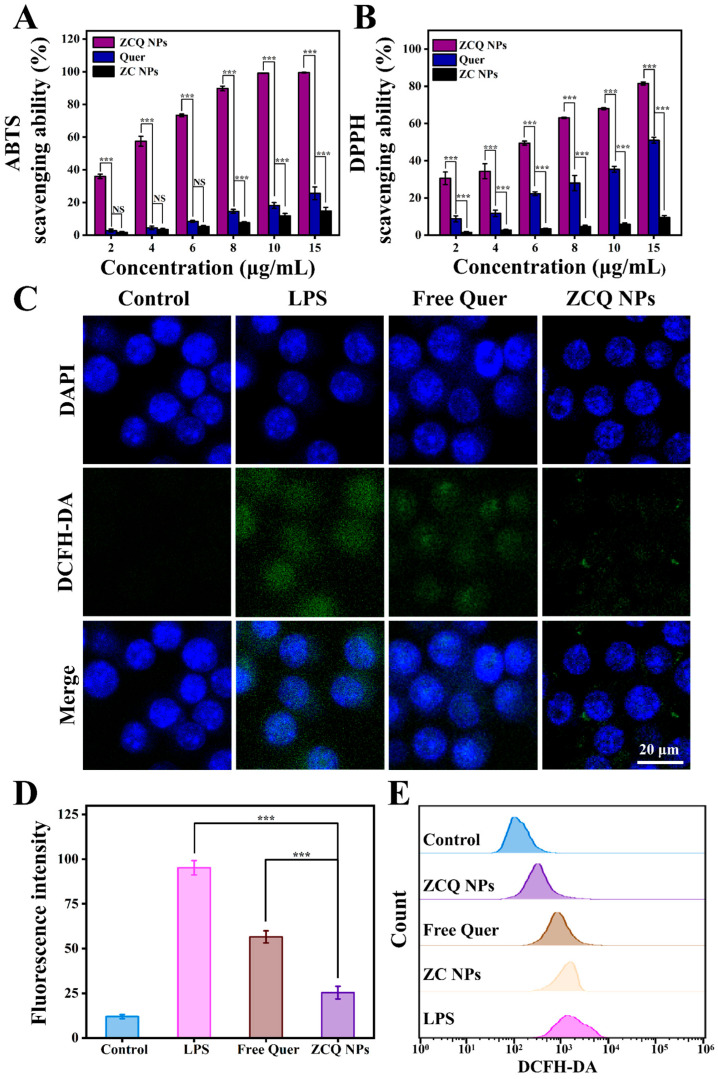
(**A**) ABTS- and (**B**) DPPH radical-scavenging activities of free Quer, ZC NPs and ZCQ NPs. (**C**) The CLSM images of intracellular ROS in LPS-activated RAW 264.7 cells treated with free Quer and ZCQ NPs. (**D**) Statistical chart of fluorescence intensity of ROS in RAW 264.7 cells. (**E**) The fluorescence intensity of ROS in RAW 264.7 cells treated with different samples was determined by flow cytometry. (NS: *p* > 0.05, *** *p* < 0.001).

Quer, which can provide hydrogen to react with free radicals, is widely utilized as an antioxidant in the food industry [45]. Thus, ABTS and DPPH radical-scavenging experiments were carried out to assess the antioxidant activity of ZCQ NPs. As illustrated in Figure 7A, both the free Quer and ZCQ NPs presented concentration-dependent ABTS-scavenging abilities. When the concentration was 15 μg/mL, the scavenging efficiency of ZCQ NPs was 3.89-fold higher than that of free Quer. Notably, ZCQ NPs showed significantly higher ABTS-scavenging efficiency than that of free Quer. The SC_50_ (the scavenging concentration needed to scavenge 50% of ABTS) of ZCQ NPs was 5.42 μg/mL, which was markedly lower than that of free Quer (15.03 μg/mL). As can be observed in Figure 7B, the DPPH radical-scavenging efficiency of ZCQ NPs and free Quer produced similar results to those obtained in the ABTS experiment. The DPPH radical-scavenging rate of ZCQ NPs reached up to 81.50% at a concentration of 15 μg/mL, whereas that of free Quer was only 51.02%. Additionally, ZC NPs (without Quer) presented negligible scavenging activity (less than 15%) in both ABTS and DPPH assays. Apparently, the improved antioxidant activity of the ZCQ NPs resulted from the encapsulation of Quer [15]. This phenomenon was rooted in the improved water dispersibility of Quer. Hence, the enhanced contact between ZCQ NPs and the medium was achieved. This enabled Quer to provide protons easily to free radicals existing in the surrounding environment [46].

### 2.11. Intracellular-ROS-Scavenging Ability

The excessive accumulation of ROS leads to oxidative stress, which poses a threat to human health. In this work, a DCFH-DA probe was applied to detect the ROS-scavenging capacity of ZCQ NPs and free Quer. As shown in Figure 7C, RAW 264.7 cells treated with LPS exhibited significant green fluorescence, indicating the successful ROS production stimulated by LPS. Cells treated with free Quer showed a slight decrease in green fluorescence compared to the LPS group, confirming the ROS-scavenging effect of Quer. Apparently, the fluorescence intensity of the ZCQ NPs group was significantly lower than that of the free Quer group, indicating enhancement in the ROS-scavenging capability of ZCQ NPs (Figure 7D). In addition, flow cytometry results (Figure 7E) demonstrated that fluorescence intensity in the ZC NPs group exhibited a minimal reduction relative to the positive control group. On the contrary, a gradual decrease in fluorescence intensity was observed across free Quer and ZCQ NPs, which was consistent with the results from CLSM analysis. This phenomenon was likely due to the fact that the ZCQ NPs showed more efficient uptake by RAW 264.7 cells compared with free Quer, leading to enhanced ROS-scavenging capability [47].

## 3. Materials and Methods

### 3.1. Materials

Quercetin (purity > 97%), CMCS, 2,2-diphenyl-1-picrylhydrazyl (DPPH), 2,2′-azino-bis (3-ethylbenzothiazoline-6-sulfonic acid) (ABTS) and potassium persulfate were acquired from Macklin Biochemical Technology Co., Ltd. (Shanghai, China). Lipopolysaccharide (LPS) was bought from Maokang Biotechnology Co., Ltd. (Shanghai, China). 2′,7′-Dichlorodihydrofluorescein diacetate (DCFH-DA) was obtained from Sigma-Aldrich Trading Co., Ltd. (Shanghai, China). Zein (purity > 90%) was bought from Beijing Solarbio Technology Co., Ltd. (Beijing, China). DMEM (Dulbecco’s modified Eagle’s medium) was bought from Thermo Fisher Scientific (Waltham, MA, USA).

### 3.2. The Preparation of Nanoparticles

#### 3.2.1. Preparation of ZC NPs

Zein/carboxymethyl chitosan nanoparticles (ZC NPs) were prepared based on a previously described anti-solvent precipitation method with minor modifications [48]. In brief, 100 mg of zein was dissolved in 5 mL of 80% (*v*/*v*) ethanol aqueous solution with stirring at 500 rpm to obtain the zein stock solution. Subsequently, 10, 20, 50, 100 and 200 mg of CMCS were separately dissolved in 15 mL of water and stirred at 500 rpm for 2 h for complete hydration. The prepared zein stock solution underwent rapid dropwise addition into the CMCS solution. Then, the pH of the system was adjusted to 6.8 with 10% acetic acid, followed by magnetic stirring for 3 h. The ethanol within the system was subsequently extracted via a rotary evaporation method. The lost liquid was replenished with deionized water to obtain the ZC NPs dispersion.

#### 3.2.2. Preparation of ZCQ NPs

A total of 10 mg of Quer was co-dissolved with 100 mg of zein in 5 mL of 80% ethanol aqueous solution. The mixture underwent magnetic stirring for 2 h to obtain the zein–Quer stock solution. The CMCS solution was prepared following the preparation method in Section 3.2.1. The obtained zein–Quer stock solution was rapidly added dropwise into the CMCS aqueous solution, followed by the acidification with 10% acetic acid. Then, the organic solvent in the mixture was removed by rotary evaporation. The lost volume was replenished with deionized water to obtain the dispersed ZCQ NPs solution. ZQ NPs (without CMCS) were obtained through the same steps as ZCQ NPs.

### 3.3. Particle Size, PDI and Zeta Potential

Mean particle diameter, PDI and zeta potential were examined via a Zetasizer (Nano ZS90, Malvern, UK). Briefly, 1 mL of freshly prepared ZCQ NPs was diluted to 10 mL with deionized water for the assay [49].

### 3.4. Determination of Quer EE and LC

The EE and LC of Quer in ZCQ NPs were determined based on previous methods with some modifications. In brief, the ZCQ NPs dispersion was centrifuged for 10 min and the precipitate obtained after centrifugation was dissolved in DMSO. UV absorbance was then tested at 380 nm to calculate the Quer content (free Quer). The weight of the total nanoparticles was determined by freeze-drying the centrifuged supernatant and subsequent weighing of the dried powder.

(1) $$\mathrm{EE}\;(\%)=\frac{total\;Quer\;input-free\;Quer}{total\;Quer\;input}\times 100$$

(2) $$\mathrm{LC}\;(\%)=\frac{total\;Quer\;input-free\;Quer}{total\;weight\;of\;nanoparticles}\times 100$$

### 3.5. Morphological Analysis

The morphology of ZCQ NPs was observed by TEM. Before observation, 200 μL of the diluted ZCQ NPs dispersion was dropped on a dedicated copper net and dried in air.

### 3.6. Ultraviolet–Visible Spectra (UV-vis)

The UV-vis spectra of ZCQ NPs and free Quer were determined by a UV-vis spectrophotometer (SP-756P, Shanghai Spectroscopic Instrument Co., Ltd., Shanghai, China). The wavelength scanning range was 250 nm to 800 nm.

### 3.7. Fourier Transform–Infrared Spectroscopy (FT-IR)

The structural characterization of zein, free Quer, CMCS and ZCQ NPs was performed using FT-IR spectrometer with a scanning range from 500 to 4000 cm^−1^, with 32 scans acquired. A 2 cm^−1^ resolution was used.

### 3.8. XRD

The crystal diffraction pattern of the ZCQ NPs was determined using an XRD (Bruker D8, Odelzhausen, Germany). The instrument was configured with a Cu Kα radiation source. The 2θ range was from 5° to 60°, and the scanning speed was 5°/min.

### 3.9. In Vitro Drug Release of ZCQ NPs

The in vitro drug-release properties of free Quer and ZCQ NPs were investigated under conditions of the SGI tract. The simulated gastric fluid (SGF) was obtained by dispersing 5 g pepsin in 500 mL deionized water, and the solution was acidified to a pH of 1.5 via 1 M HCl. The simulated intestinal fluid (SIF) was prepared by dissolving potassium dihydrogen phosphate and trypsin in deionized water, and then the pH value of the solution was optimized to 6.8 via NaOH.

A 2 mL sample was sealed in a 3500 Da molecular-truncated dialysis bag and incubated in a conical flask containing 25 mL of SGF while shaking at 100 rpm for 2 h at 37 °C. A 2 mL digestion sample was collected at 30 min intervals and supplemented with the same volume of SGF to maintain digestion conditions. Subsequently, the gastric juice digestion was stopped, the dialysis bag was transferred to 25 mL of SIF, and the shaking digestion was continued at 37 °C and 100 rpm for 4 h. Digestion samples were collected every 30 min and supplemented with the same volume of SIF to maintain digestion conditions. The content of Quer in the digested samples was detected by UV spectrophotometry at a wavelength of 380 nm.

### 3.10. Stability Investigation

#### 3.10.1. Effect of Ionic Strength

To assess the impact of the change in ionic strength of the system on the ZCQ NPs, the freshly prepared ZCQ NPs and ZQ NPs dispersions were diluted with an equal volume of NaCl solution with concentrations of 20, 40, 60, 80 and 100 mM, respectively. Then the mixture was vortexed for 10 min before standing for 24 h. Subsequently, the particle size was determined.

#### 3.10.2. Photostability of Quer

The photostability was evaluated via a dark-box UV analyzer (16 W, 254 nm). By exposing dispersions of free Quer, ZCQ NPs and ZQ NPs under the dark-box UV analyzer at 15, 30, 45, 60, 75 and 90 min, the remaining amount of Quer in the dispersions was measured using UV–visible spectrophotometry at 380 nm, and the retention values were calculated. The retention rate of Quer was determined by the following formula:

(3) $$\mathrm{The}\;\mathrm{retention}\;\mathrm{rate}\;\mathrm{of}\;\mathrm{Quer}=\frac{A_{t}}{A_{0}}$$

where A_0_ and A_t_ were the initial absorbance of Quer and the absorbance at different time points, respectively.

#### 3.10.3. Thermal Stability

To evaluate the effect of heating on the system, the dispersions of free Quer, ZCQ NPs and ZQ NPs were heated at 65, 75 and 85 °C, for 30 min, respectively. Subsequently, the particle size of nanoparticles was measured. In addition, to deeply compare the stability differences between ZCQ NPs, ZQ NPs and free Quer, the amount of Quer remaining in free Quer, ZCQ NPs and ZQ NPs dispersions was determined. The retention rate of Quer was determined by the following formula:

(4) $$\mathrm{The}\;\mathrm{retention}\;\mathrm{rate}\;\mathrm{of}\;\mathrm{Quer}=\frac{A_{h}}{A_{0}}$$

where A_0_ and A_h_ were the initial absorbance of Quer and the absorbance at 380 nm after carrying out different thermal experiments, respectively.

#### 3.10.4. Storage Stability

Freshly prepared ZCQ NP dispersions were stored at 4 °C, and their particle size and PDI were tested at 0, 5, 10, 15, 20, 25 and 30 days. To exclude the influence of light on the results, the entire storage process was shielded from light.

### 3.11. Redispersion of Nanoparticle

The freshly synthesized ZCQ NPs and ZQ NPs were freeze-dried for a duration of 36 h. The powders obtained were then redispersed in 25 mL of deionized water for 3 h. Visual observation of the dissolution and precipitation characteristics of the redispersed nanoparticles was performed, and images were captured. Moreover, DLS and zeta potential measurements were utilized for comparative analysis.

### 3.12. Cellular Uptake

The uptake of Caco-2 cells was analyzed via confocal laser scanning microscopy (CLSM, FV 3000, Olympus Corporation, Tokyo, Japan). Fluorescein isothiocyanate (FITC) was substituted for Quer to investigate the effect of the formation of ZC NPs (FITC-encapsulated ZC NPs, F@ZC NPs) on the cellular-drug-uptake capability. Caco-2 cells at a density of 2 × 10^6^ cells/dish were seeded onto the confocal dishes and then cultured (37 °C, 5% CO_2_) to allow the cells to adhere fully. Then, 1 mL of the free FITC or F@ZC NPs dispersion was added to each confocal dish and incubated for 3 and 6 h, respectively. After the incubation period, the cells were fixed with 4% paraformaldehyde fixative solution and stored at 4 °C for half an hour. Then, 100 μL of 4′,6-diamidino-2-phenylindole (DAPI) was added to each confocal dish after fixation. After staining for 15 min, images were captured using CLSM.

For a better comparison of the cellular uptake of free FITC and F@ZC NPs, quantitative analysis was also performed via flow cytometry.

### 3.13. Endocytosis Mechanisms of Nanoparticles

To clarify the cellular-uptake mechanism of nanoparticles, different endocytosis inhibitors were employed. Caco-2 cells were seeded into 6-well plates and cultured at 37 °C under 5% CO_2_ to establish the uptake model. Subsequently, cells were pre-treated with amiloride (10 μg/mL), chlorpromazine (10 μg/mL), and indomethacin (10 μg/mL) for 0.5 h, respectively. Following incubation, the cells were treated with a F@ZC NPs dispersion for 3 h. Upon completion of the incubation, the cells were collected, and their fluorescence intensity was analyzed using flow cytometry. The 4 °C treatment group was included to assess the role of energy in nanoparticle uptake.

### 3.14. Antioxidant Activity

#### 3.14.1. ABTS-Scavenging Assay

In brief, a reserve solution of 7.40 mM ABTS and a 2.45 mM K_2_S_2_O_8_ solution were prepared. The ABTS working liquid was obtained by mixing the above two substances in equal volume and reacting under darkness for 16 h. The working liquid was diluted with ethanol so that its absorbance was 0.70 ± 0.02 at 734 nm. Then, the sample solution was mixed with ABTS working solution, and the reaction was carried out in the dark for 10 min. Subsequently, the UV absorbance was measured at 734 nm. Ultrapure water was used instead of the sample solution as a control. The following formula was employed to calculate the ABTS free radical-scavenging ability:

(5) $$\mathrm{ABTS-scavenging}\;\mathrm{ability}\;(\%)=(1-\frac{A_{S}}{A_{C}})\times 100$$

where *A_S_* and *A_C_* were the absorbance of the samples and control, respectively.

#### 3.14.2. DPPH-Scavenging Assay

DPPH powder was dissolved in ethanol to obtain the DPPH solution (0.1 mM). The sample solution was incubated with the DPPH solution in a 1:1 volume ratio and kept at room temperature in the dark for half an hour. Deionized water was used instead of the sample solution as a control. After incubation, the absorbance of the samples was detected at 517 nm. The formula below was used to calculate DPPH radical-scavenging ability:

(6) $$\mathrm{DPPH-scavenging}\;\mathrm{ability}\;(\%)=(1-\frac{A_{S}}{A_{C}})\times 100$$

where *A_S_* and *A_C_* were the absorbance of the samples and control, respectively.

### 3.15. Intracellular-ROS-Scavenging Activity

RAW 264.7 cells were inoculated onto confocal dishes. And LPS was utilized to stimulate ROS production. Subsequently, the cells were cultured with free Quer and ZCQ NP dispersion for 24 h. Following the drug treatment, PBS was utilized for washing the cells. Then, 1 mL of DCFH-DA solution was added to each plate and incubated for half an hour at 37 °C. After the incubation, the ROS level was observed under CLSM at 488 nm.

Further assessment of the ROS-scavenging capability of ZCQ NPs was conducted using flow cytometry. RAW 264.7 cells were seeded on a 12-well plate and induced with LPS to generate ROS. Then, RAW 264.7 cells were treated with 20 μg/mL of free Quer and ZCQ NPs dispersion for 24 h. In addition, ZC NPs group was included to further assess the impact of zein and CMCS on the ROS-scavenging activity of Quer. Subsequently, the drug solution was discarded, and the DCFH-DA solution was added. The fluorescence intensity was determined using flow cytometry.

### 3.16. Statistical Analysis

The experiments in this study were performed in triplicate, with data presented as mean ± standard deviation. Statistical analysis of significant differences was performed using Graph Pad Prism 9.5.0.

## 4. Conclusions

In this study, ZCQ NPs were successfully synthesized using the anti-solvent precipitation method. The obtained ZCQ NPs exhibited a spherical shape with uniform morphology, appropriate particle size, ideal zeta potential and high EE. FT-IR demonstrated that the formation of ZCQ NPs was predominantly driven by hydrophobic interactions and hydrogen bonding. Results of XRD tests verified that Quer was encapsulated in ZCQ NPs in an amorphous state. Additionally, the prepared ZCQ NPs displayed excellent redispersibility and enhanced stability in terms of light, heat and ionic strength compared to ZQ NPs. ZCQ NPs could also maintain splendid stability under long-term storage conditions. In addition, the formation of ZC NPs markedly enhanced the cellular-uptake ability of the drug encapsulation inside. And the antioxidant capacity of encapsulated Quer was obviously improved. In conclusion, ZCQ NPs can serve as an ideal drug-delivery carrier for improving the stability and antioxidant capacity of Quer.

## Data Availability

The original contributions presented in this study are included in the article. Further inquiries can be directed to the corresponding author.

## References

[1] Liu L., Barber E., Kellow N.J., Williamson G. (2025). Improving quercetin bioavailability: A systematic review and meta-analysis of human intervention studies. Food Chem..

[2] Lin J.C., Li F.Y., Jiao J.Z., Qian Y.H., Xu M., Wang F., Sun X.H., Zhou T., Wu H.L., Kong X.N. (2025). Quercetin, a natural flavonoid, protects against hepatic ischemia- reperfusion injury via inhibiting Caspase-8/ASC dependent macrophage pyroptosis. J. Adv. Res..

[3] Liu Q., Liu P., Ban Q. (2025). Green development strategy for efficient quercetin-loaded whey protein complex: Focus on quercetin loading characteristics, component interactions, stability, antioxidant, and in vitro digestive properties. Food Chem..

[4] Wang W., Cui Y., Liu X., Zheng M.Z., Wang Y.H., Liu J.S., Liu H.M. (2025). A novel zein/Auricularia cornea Ehrenb polysaccharides coated quercetin nanocarrier: Characterization, stability, antioxidant activity, and bioaccessibility. Colloids Surf. A.

[5] Yang J., Shen M., Wen H., Luo Y., Huang R., Rong L., Xie J. (2020). Recent advance in delivery system and tissue engineering applications of chondroitin sulfate. Carbohydr. Polym..

[6] Yu F., Luo H., Wang Y., Wei Z., Li B., Zhao Y., Wu P., Wang J., Yang H., Gao J. (2024). Preparation of curcumin-loaded chitosan/lecithin nanoparticles with increased anti-oxidant activity and in vivo bioavailability. Int. J. Biol. Macromol..

[7] Glusac J., Fishman A. (2021). Enzymatic and chemical modification of zein for food application. Trends Food Sci. Technol..

[8] Yu Y.B., Wang C., Chen T.T., Wang Z.W., Yan J.K. (2021). Enhancing the colloidal stabilities of zein nanoparticles coated with carboxylic curdlans. Lwt-Food Sci. Technol..

[9] Ghorbani M., Mahmoodzadeh F., Maroufi L.Y., Nezhad-Mokhtari P. (2020). Electrospun tetracycline hydrochloride loaded zein/gum tragacanth/poly lactic acid nanofibers for biomedical application. Int. J. Biol. Macromol..

[10] Huang Y., Zhan Y., Luo G., Zeng Y., McClements D.J., Hu K. (2023). Curcumin encapsulated zein/caseinate-alginate nanoparticles: Release and antioxidant activity under in vitro simulated gastrointestinal digestion. Curr. Res. Food Sci..

[11] Xu X., Dai D., Yan H., Du J., Zhang Y., Chen T. (2025). Enhancing mechanical and blocking properties of gelatin films using zein-quercetin nanoparticle and applications for strawberry preservation. Food Chem..

[12] Sha L., Raza H., Jia C., Khan I.M., Yang H., Chen G. (2025). Genipin-enriched chitosan-Zein nanoparticles for improved curcumin encapsulation. Int. J. Biol. Macromol..

[13] Zhang T., Yu S., Tang X., Ai C., Chen H., Lin J., Meng H., Guo X. (2022). Ethanol-soluble polysaccharide from sugar beet pulp for stabilizing zein nanoparticles and improving encapsulation of curcumin. Food Hydrocoll..

[14] Dong Y., Wei Z., Wang Y., Tang Q., Xue C., Huang Q. (2022). Oleogel-based Pickering emulsions stabilized by ovotransferrin–carboxymethyl chitosan nanoparticles for delivery of curcumin. Lwt-Food Sci. Technol..

[15] Zhang H., Fu Y.Y., Niu F.G., Li Z.Y., Ba C.J., Jin B., Chen G.W., Li X.M. (2018). Enhanced antioxidant activity and in vitro release of propolis by acid-induced aggregation using heat-denatured zein and carboxymethyl chitosan. Food Hydrocoll..

[16] Fang M., Wang J., Fang S., Zuo X. (2023). Fabrication of carboxymethyl chitosan films for cheese packaging containing gliadin-carboxymethyl chitosan nanoparticles co-encapsulating natamycin and theaflavins. Int. J. Biol. Macromol..

[17] Liu C.Z., Yuan Y.K., Ma M.J., Zhang S.Z., Wang S.H., Li H., Xu Y., Wang D.F. (2020). Self-assembled composite nanoparticles based on zein as delivery vehicles of curcumin: Role of chondroitin sulfate. Food Funct..

[18] Zhang D., Jiang F., Ling J., Ouyang X.K., Wang Y.G. (2021). Delivery of curcumin using a zein-xanthan gum nanocomplex: Fabrication, characterization, and in vitro release properties. Colloids Surf. B Biointerfaces.

[19] Yu Y.-B., Wu M.-Y., Wang C., Wang Z.-W., Chen T.-T., Yan J.-K. (2020). Constructing biocompatible carboxylic curdlan-coated zein nanoparticles for curcumin encapsulation. Food Hydrocoll..

[20] Wu J., Chen J., Wei Z., Zhu P., Li B., Qing Q., Chen H., Lin W., Lin J., Hong X. (2023). Fabrication, Evaluation, and Antioxidant Properties of Carrier-Free Curcumin Nanoparticles. Molecules.

[21] Liu J., Yu H., Kong J., Ge X., Sun Y., Mao M., Wang D.Y., Wang Y. (2024). Preparation, characterization, stability, and controlled release of chitosan-coated zein/shellac nanoparticles for the delivery of quercetin. Food Chem..

[22] Xiao J., Nian S., Huang Q. (2015). Assembly of kafirin/carboxymethyl chitosan nanoparticles to enhance the cellular uptake of curcumin. Food Hydrocoll..

[23] Ge Q., Rong S., Yin C., McClements D.J., Fu Q., Li Q., Han Y., Liu F., Wang S., Chen S. (2024). Calcium ions induced ι-carrageenan-based gel-coating deposited on zein nanoparticles for encapsulating the curcumin. Food Chem..

[24] Jun Y., Jieqiong L., Xianxiang C., Liyuan R., Mingyue S., Yuanxing W., Jianhua X. (2022). Mesona chinensis polysaccharide/zein nanoparticles to improve the bioaccesibility and in vitro bioactivities of curcumin. Carbohydr. Polym..

[25] Wang M., Fu Y., Chen G., Shi Y., Li X., Zhang H., Shen Y. (2018). Fabrication and characterization of carboxymethyl chitosan and tea polyphenols coating on zein nanoparticles to encapsulate β -carotene by anti-solvent precipitation method. Food Hydrocoll..

[26] Roy V.C., Razzak M.A., Ho T.C., Surendhiran D., Park J.S., Chun B.S. (2022). Fabrication of zein and j-carrageenan colloidal particles for encapsulation of quercetin: In-vitro digestibility and bio-potential activities. J. Ind. Eng. Chem..

[27] Zhou J.F., Zheng G.D., Wang W.J., Yin Z.P., Chen J.G., Li J.E., Zhang Q.F. (2021). Physicochemical properties and bioavailability comparison of two quercetin loading zein nanoparticles with outer shell of caseinate and chitosan. Food Hydrocoll..

[28] Sharma J.B., Bhatt S., Saini V., Kumar M. (2021). Pharmacokinetics and Pharmacodynamics of Curcumin-Loaded Solid Lipid Nanoparticles in the Management of Streptozotocin-Induced Diabetes Mellitus: Application of Central Composite Design. Assay Drug Dev. Technol..

[29] Xue J.L., Zhang Y.Q., Huang G.R., Liu J., Slavin M., Yu L.L. (2018). Zein-caseinate composite nanoparticles for bioactive delivery using curcumin as a probe compound. Food Hydrocoll..

[30] Paliwal R., Palakurthi S. (2014). Zein in controlled drug delivery and tissue engineering. J. Control. Release.

[31] Liu Q., Jing Y., Han C., Zhang H., Tian Y. (2019). Encapsulation of curcumin in zein/caseinate/sodium alginate nanoparticles with improved physicochemical and controlled release properties. Food Hydrocoll..

[32] Dai L., Li R., Wei Y., Sun C., Mao L., Gao Y. (2018). Fabrication of zein and rhamnolipid complex nanoparticles to enhance the stability and in vitro release of curcumin. Food Hydrocoll..

[33] Lin M., Fang S., Zhao X., Liang X., Wu D. (2020). Natamycin-loaded zein nanoparticles stabilized by carboxymethyl chitosan: Evaluation of colloidal/chemical performance and application in postharvest treatments. Food Hydrocoll..

[34] Khan M.A., Zhou C.F., Zheng P., Zhao M., Liang L. (2021). Improving Physicochemical Stability of Quercetin-Loaded Hollow Zein Particles with Chitosan/Pectin Complex Coating. Antioxidants.

[35] Chen S., Han Y.H., Sun C.X., Dai L., Yang S.F., Wei Y., Mao L.K., Yuan F., Gao Y.X. (2018). Effect of molecular weight of hyaluronan on zein-based nanoparticles: Fabrication, structural characterization and delivery of curcumin. Carbohydr. Polym..

[36] Qiu C., Wang B., Wang Y., Teng Y. (2017). Effects of colloidal complexes formation between resveratrol and deamidated gliadin on the bioaccessibility and lipid oxidative stability. Food Hydrocoll..

[37] Zhang Z.H., Hu Y., Ji H.Y., Lin Q.Z., Li X.J., Sang S.Y., McClements D.J., Chen L., Long J., Jiao A.Q. (2023). Physicochemical stability, antioxidant activity, and antimicrobial activity of quercetin-loaded zein nanoparticles coated with dextrin-modified anionic polysaccharides. Food Chem..

[38] Zhang Z., Li X., Sang S., McClements D.J., Chen L., Long J., Jiao A., Jin Z., Qiu C. (2023). Preparation, properties and interaction of curcumin loaded zein/HP-β-CD nanoparticles based on electrostatic interactions by antisolvent co-precipitation. Food Chem..

[39] Niloofar G.-O., Ahmad A., Marzieh M. (2022). Fabrication, characterization and in vitro cell exposure study of zein-chitosan nanoparticles for co-delivery of curcumin and berberine. Int. J. Biol. Macromol..

[40] Yuan Y., Xiao J., Zhang P., Ma M., Wang D., Xu Y. (2021). Development of pH-driven zein/tea saponin composite nanoparticles for encapsulation and oral delivery of curcumin. Food Chem..

[41] Ma J.J., Yu Y.G., Yin S.W., Tang C.H., Yang X.Q. (2018). Cellular Uptake and Intracellular Antioxidant Activity of Zein/Chitosan Nanoparticles Incorporated with Quercetin. J. Agric. Food Chem..

[42] Wang Y., Pi C., Feng X., Hou Y., Zhao L., Wei Y. (2020). The Influence of Nanoparticle Properties on Oral Bioavailability of Drugs. Int. J. Nanomed..

[43] Tao J., Diao L., Chen F.C., Shen A., Wang S.T., Jin H.Y., Cai D.W., Hu Y. (2021). pH-Sensitive Nanoparticles Codelivering Docetaxel and Dihydroartemisinin Effectively Treat Breast Cancer by Enhancing Reactive Oxidative Species-Mediated Mitochondrial Apoptosis. Mol. Pharm..

[44] Dai Z.J., Yin W.T., Li J.H., Ma L.J., Chen F., Shen Q., Hu X.S., Xue Y., Ji J.F. (2025). Zein and Trimethyl Chitosan-Based Core-Shell Nanoparticles for Quercetin Oral Delivery to Enhance Absorption by Paracellular Pathway in Obesity Mice. Biomater. Res..

[45] Costa L.G., Garrick J.M., Roquè P.J., Pellacani C. (2016). Mechanisms of Neuroprotection by Quercetin: Counteracting Oxidative Stress and More. Oxid. Med. Cell. Longev..

[46] Chen S., Li Q., McClements D.J., Han Y., Dai L., Mao L., Gao Y. (2020). Co-delivery of curcumin and piperine in zein-carrageenan core-shell nanoparticles: Formation, structure, stability and in vitro gastrointestinal digestion. Food Hydrocoll..

[47] Sorasitthiyanukarn F.N., Muangnoi C., Thaweesest W., Rojsitthisak P., Rojsitthisak P. (2019). Enhanced cytotoxic, antioxidant and anti-inflammatory activities of curcumin diethyl disuccinate using chitosan-tripolyphosphate nanoparticles. J. Drug Deliv. Sci. Technol..

[48] Meng R., Wu Z., Xie Q.-T., Cheng J.-S., Zhang B. (2021). Preparation and characterization of zein/carboxymethyl dextrin nanoparticles to encapsulate curcumin: Physicochemical stability, antioxidant activity and controlled release properties. Food Chem..

[49] Mariano A., Li Y.O., Singh H., McClements D.J., Davidov-Pardo G. (2024). Encapsulation of orange-derived hesperetin in zein/pectin nanoparticles: Fabrication, characterization, stability, and bioaccessibility. Food Hydrocoll..

